# Clinical management of lung cancer patients during the outbreak of COVID-19 epidemic

**DOI:** 10.1186/s13027-020-00322-7

**Published:** 2020-09-23

**Authors:** Li Wang, Man Jiang, Jialin Qu, Na Zhou, Xiaochun Zhang

**Affiliations:** Precision Medicine Center of Oncology, The Affiliated Hospital of Qingdao University, Qingdao University, 16 Jiangsu Road, Qingdao, 266003 China

**Keywords:** COVID-19, Lung cancer, Patient management

## Abstract

The rapid growth of 2019 novel coronavirus (COVID-19) outbreak in Wuhan, China, at the early December 2019. COVID-19 spread all over the word just a few months. The outbreak of COVID-19 infection poses major threat to international health and economy. World Health Organization (WHO) announced that the new coronavirus was an international public health emergency on January 30, 2020. However, with the spread of COVID-19, the routine medical care of lung cancer patients was affected. Because lung cancer patients have low immunity after anti-tumor treatment, they should become the main targets for epidemic prevention. Lung cancer patients are increasingly concerned about the prevention of COVID-19. It is necessary to provide individualized medical treatment and management for lung cancer patients based on patients’ conditions and regional epidemic patterns.

## Introduction

Coronaviruses (CoVs) are enveloped non-segmented positive sense RNA viruses, belongs to the subfamily Coronavirinae, the order Nidovirales, and this subfamily including alpha-, beta-, gamma-, and delta-coronavirus [1]. Coronaviruses mainly cause infections in birds and mammals and, in recent decades, also have shown an ability to infecting humans [2]. The outbreak of beta- coronavirus including severe acute respiratory syndrome coronavirus (SARS) in 2002 [3] and Middle East respiratory syndrome coronavirus (MERS) in 2012 [4] has confirmed that the lethality of coronaviruses when they infect humans. The mortality rates of SARS and MERS are 10 and 37%, respectively [5, 6].

At the end of 2019, a novel influenza coronavirus (COVID-19) that similar to SARS and MERS appeared in Wuhan, Hubei, China and has been confirmed that has the ability of human-to-human transmission [7–9]. The genome of COVID-19 is a single-stranded positive sense RNA [10]. The gene sequence analysis showed that COVID-19 has a typical coronavirus genome structure and belongs to the beta- coronavirus cluster [10]. With the rapid increase of the number of cases and increasing evidence of human-to-human transmission, the virus is more contagious than SARS and MERS [11–16]. Moreover, COVID-19 show very different virological characteristics compare with SARS. For the SARS, the level of RNA usually reaches a peak after 7–10 days of symptoms. However, the level of RNA reaches a peak within 5 days of the symptoms of COVID-19, which can be 1000 times higher than the SARS [17]. In additional, three major COVID-19 variants have been found, which are divided into A, B and C types. Among them, the A virus is the closest to the coronavirus found in bats and pangolins. It is the original virus type and the B type is derived from Type A, Type C are derived from Type B. Nearly half of the samples infected with Type A are from outside East Asia, mainly in the United States and Australia, and two-thirds of American samples are infected with Type A, and Type B is the most common in East Asia. type C is the main virus type spread in Europe [18].

Lung cancer was the most incident and lethal malignant tumor among all human cancers, with an estimated that there are about 234,030 new cases with lung cancer in the United States in 2018 [19]. There are a large number of lung cancer patients. With the prevalence of COVID-19 epidemic, the routine medical care of lung cancer patients was affected. Moreover, lung cancer patients are more susceptible to COVID-19 since they are usually elderly patients and have low immunity and poor lung conditions. Thus, they need to be well protected from COVID-19.

To minimize the effect of the epidemic, providing clinical management for lung cancer patients in the global epidemic scenario is an urgent requirement. In this review, we focus on the epidemiological characteristics, early diagnosis, patient management and mental health of lung cancer patients during the COVID-19 epidemic.

## Molecular mechanism

The Coronavirus consists of nucleocapsid protein (N protein), Spike protein (S protein), small envelope glycoprotein (E protein), membrane glycoprotein (M protein), Hemagglutinin-esterase dimer (HE protein) and RNA. The spike protein of coronaviruses facilitates viral entry into target cells. The spike protein is a type I transmembrane glycoprotein and can be divided into two functional units: the receptor binding subunit S1 and the membrane fusion subunit S2 [20]. S1 including two domains: the C-terminal receptor binding domain (RBD) domain and the N-terminal domain [21]. S1 facilitates virus infection by binding to host receptors. And S2 is responsible for the fusion of the virus and the membrane of host cell. COVID-19 uses the serine protease TMPRSS2 as an initiator of spike protein [22].

Angiotensin-converting enzyme 2 (ACE2) is a type I membrane protein mainly expressed in the lung, heart, kidney and intestine [23–25]. ACE2 consists of an N-terminal peptidase domain (PD) and a C-terminal Collectrin-like domain (CLD) [23, 26]. The PD of ACE2 mainly interacts with the RBD domain of the S protein of the coronavirus [27–31]. And the RBD domain of the S protein is recognized by the extracellular PD of ACE2 mainly through polar residues [32]. The structural information of ACE2 is limited to the PD domain. So, the S protein of COVID-19 exploit ACE2 for host infection [33–36].

COVID-19 invade the human body by combine to ACE2 receptor, resulting in excessive activation of T cells, and producing large amounts of pro-inflammatory cytokines and chemokines such as IL-6, GM-CSF. It also generates a positive feedback mechanism with monocytes, further produce large amounts of IL-6 and other cytokines, and then emerge a cytokine storm [14, 37]. The cytokine storm will trigger a violent attack by the immune system to the human body, cause ARDS and multiple organ failure, and finally lead to death in severe cases of COVID-19 infection.

## Epidemiological

Based on current epidemiological investigations, the COVID-19 of median incubation period is 4.75 days (interquartile range: 3.0–7.2) days [38]. The main route transmission of COVID-19 is through respiratory droplets and close contact. A study found viruses in chairs, bed rails, glass windows, floors, light switches and toilets in patient rooms. However, the results of the air samples were negative, but the samples collected at the exhaust ports were positive, indicating that the air, surface environmental, and personal protective equipment contamination is a potential medium of transmission [39].

Common symptoms are fever, cough, and myalgia or fatigue [40]. Less common symptoms were sputum production, headache, haemoptysis, and diarrhoea. About 55% of patients experienced dyspnea (median time from illness onset to dyspnoea was 8.0 days [IQR 5 · 0–13-1 · 0]) [40]. Even some cases show loss of taste and smell [17]. Approximately 25.5, 69.9, and 4.5% of patients were diagnosed with severe pneumonia, mild pneumonia, and non-pneumonia, respectively [38]. Laboratory features of patients usually include leukopenia, lymphopenia and elevated aspartate aminotransferase. Compared with ICU patients, non-ICU patients had lower plasma levels of IL2, IL7, IL10, GSCF, IP10, MCP1, MIP1A, and TNFα [14]. COVID-19 pneumonia baseline chest CT presented with ground glass opacities (72%), consolidation (13%), crazy paving pattern (12%), interlobular thickening (37%), adjacent pleura thickening (56%), and linear opacities combined (61%) [41]. The COVID-19 deaths are more common in the majority of patients over 60 years age and suffer from underlying diseases such as hypertension, cardiovascular disease and cancer [42].

### Epidemiological characteristics of COVID-19 with neoplasm

Chinese center for disease control and prevention [40] has shown that 72,314 cases of pneumonia were reported, including confirmed cases (61.8%), suspected cases (22.4%), clinically diagnosed cases (14.6%), and asymptomatic infections (1.2%). About 107 (0.5%) were cancer patients, of which 6 deaths, and the crude mortality rate was 5.6%, which was higher than the overall population crude mortality rate of 2.3% [40]. A study [43] analyzed 1590 patients with COVID-19 as of January 31, 2020, 18 (1%) had a history of cancer, 28% of cancer patient were lung cancer patients. Cancer patients have a higher risk of serious events than non-cancer patients (39 and 8%, *P* = 0.000,3), and symptoms deteriorate more rapidly.

### Early identification of COVID-19 pneumonia in lung cancer patients

The symptoms of lung cancer patients include cough, sputum, dyspnea, and fever. After anti-tumor therapy, patients may have various treatment-related side effects. Moreover, the imageology detection of lung cancer is not typical due to influence of the tumor. Therefore, it is very difficult to identify and diagnose the COVID-19 pneumonia early. It is necessary to actively improve the relevant examinations, carry out differential diagnosis, and clarify the cause.

The diagnosis of COVID-19 pneumonia needs to be combined with the epidemiological history and clinical manifestations (① fever and / or respiratory symptoms; ② with imaging features of COVID-19 pneumonia; ③ normal or reduced white blood cell count or decreased lymphocyte count in the early stage of the disease).

#### General virus detection

##### Cell culture

Traditional cell isolation and culture is a process of obtaining a large number of cells by simulating the in vivo environment in vitro. This method is the gold standard for virus detection. However, the training cycle is long and the sensitivity is low, and it cannot be used for rapid diagnosis. Therefore, it is not recommended as the first detection method [44].

##### Serological testing

Currently, serological tests include immunochromatographic methods, enzyme-linked immunosorbent assays, and more. Detection the antigens by collecting patient specimens that specifically bind to known antibodies. The serological testing Kit are easy to standardize and commercialize. It has the characteristics of convenient and fast operation, and suitable for screening of hidden infections and large-scale people. Serological tests often show these symptoms including, leukopenia, absolute lymphocyte reduction, abnormal liver function, coagulation function. A study developed a rapid and simple way which can detect IgM and IgG antibodies simultaneously against COVID-19 in human blood within 15 min which can detect patients at different infection stages [45].

##### Nucleic acid detection

The characteristics of nucleic acid detection are high sensitivity and strong specificity. It has become the mainstream method for novel coronavirus detection. Compared with other methods, the nucleic acid detection requires less sample and can be used for high-quality single-cell transcriptome sequencing. Currently, nucleic acid detection is a confirmatory indicator in China.

##### Real-time RT-PCR

Real-time RT-PCR is widely deployed in diagnostic virology. In acute respiratory infection, RT-PCR is routinely used to detect causative viruses from respiratory secretions [46]. A study [47] successfully detects COVID-19, and further discriminates COVID-19 from SARS-CoV by this method. And this study gives the detection primer sequence and determination method. This provides a theoretical basis for the detection of 2019-nCov.

##### Chest CT examination

Chest CT examination plays an important role in the initial diagnosis of COVID-19 pneumonia. Multiple patchy ground glass opacities in bilateral multiple lobular with periphery distribution are typical chest CT imaging features of the COVID-19 pneumonia [41].

#### Differential diagnosis of lung cancer patients

For lung cancer patients, it is very important to make a differential diagnosis and fully assess whether the lung cancer patients have other possibilities of causing fever and respiratory symptoms.

##### Radiation pneumonia

Radiation pneumonia can occur in lung cancer patients 1 month to 3 months after radiotherapy, and some case occur during radiotherapy. Patients may have symptoms such as fever, dry cough, etc. Chest CT showed ground glass and flaky lung shadows [48]. Moreover, with the improvement of radiotherapy technology, the imaging characteristics of radiation pneumonitis have also become unspecific. This makes it difficult to distinguish radiation pneumonia from COVID-19 pneumonia. We need a multidisciplinary team in the radiotherapy department, imaging department, oncology department, and infection department to fully analyze the radiotherapy time, radiotherapy dose, radiotherapy site, and imaging characteristics, and carefully identify the patient’s specific clinical manifestations and test results.

##### Immune checkpoint inhibitor-associated pneumonia

There is a risk of immune checkpoint inhibitor-associated pneumonia in patients who receiving immunotherapy. The clinical symptoms of the immune checkpoint inhibitor-associated pneumonia including fever, increased cough and sputum, and increased dyspnea. Chest CT shows ground glass and flake shadows in the lungs, and some patients can simply show ground glass shadows in the lungs [49]. This also requires multidisciplinary cooperation including oncology, imaging, and infectious diseases, comprehensive analysis and identification, and guidance for subsequent treatment.

##### Tumor progression

Tumor progression may lead to obstructive pneumonia, cancerous lymphadenitis, pleural effusion increased, pericardial effusion, etc., which can lead to fever and respiratory symptoms. Chest and abdomen CT and tumor markers can assist in differential diagnosis.

## Regular examination of lung cancer patients

Patients with regular examination after lung cancer surgery can delay the examination if the condition is stable. For advanced lung cancer patients with targeted therapy or immunotherapy, the scheduled examination can be postponed or postponed according to the cancer status. The interval between examinations can be extended to more than 3 months for patients with symptomatic remission or stable disease. It is recommended that patients continue to take the original medication and closely monitor the signs during the postponed examination period. Patients should be examined admitted to the hospital for treatment, if symptoms progressively worsen. And the examination procedures and methods should be simplified to shorten hospital stays. Patients may consult oncologists online or offline to understand the condition after examination.

## Clinical management

### The treatment of general patient

#### Antiviral treatments

Antiviral treatments have been shown by clinical observation and research to have certain effects in COVID-19 therapy. According to China’s New Coronavirus Pneumonia Diagnosis and Treatment Plan (Trial Version 8), drugs with potential antiviral effects should be used early in the course of the disease, and it is recommended to focus on patients with high risk factors for severe illness patients. Lopinavir/ritonavir and ribavirin alone are not recommended for COVID-19 patients.

##### Lopinavir and ritonavir

Lopinavir and ritonavir is widely used as a boosted protease inhibitor in the treatment of HIV infection [50]. Lopinavir and ritonavir can block the enzymes that required for virus replication. Lopinavir is often combined with ritonavir to extend the half-life of lopinavir by the inhibition of cytochrome P450 [51]. A study had found that the Lopinavir and ritonavir combined with ribavirin in the treatment of SARS was associated with better effect [52]. And animal studies show that Lopinavir and Ritonavir can reduce levels of coronavirus that cause SARS and MERS [53].

##### Remdesivir

Remdesivir, a nucleotide analog GS-5734 that produced by biotechnology company Gilead, has a certain anti-coronal virus effect in animal experiments [54]. In addition, Remdesivir can improve lung function, reduce lung viral load and severe lung pathology in mice [51]. A study reported that an American patient with COVID-19 survived by treated with Remdesivir [55]. At the same time, a study showed that 68% of the 53 patients with severe and critical new coronavirus have relieved symptoms and the mortality rate is 13% after the use of Remdesivir [56]. However, the study was conducted under sympathetic medication, and there was no control group, so it was not possible to evaluate the direct relationship between Remdesivir and patients’ symptom improvement. Although the test results released a certain positive signal, the test has limitations and still needs follow-up discussion.

#### Chloroquine and Hydroxychloroquine

Chloroquine, a widely used antimalarial and autoimmune disease drug, has been reported may be a potential broad-spectrum antiviral drug [57, 58]. Chloroquine blocks viral infection by increasing endosomal pH required for virus fusion, and interfering with the glycosylation of SARS-CoV cellular receptor [59]. A study showed that remdesivir and chloroquine are highly effective in the control of COVID-19 infection in vitro [60]. Therefore, the efficacy of Chloroquine needs to be clarified and applied to patients as soon as possible.

Hydroxychloroquine is a derivative of chloroquine. The two structures are similar and the mechanism of action is very similar, but they differ in terms of safety and tolerability in clinical use. A study showed that the temperature recovery time and cough remission time of patients were significantly shortened in hydroxychloroquine group. The proportion of patients with improved pneumonia was 80.6%, which was higher than that of the control group (54.8%) [61]. Hydroxychloroquine can significantly shorten the clinical recovery time of patients with new coronavirus and promote the improvement of pneumonia. However, hydroxychloroquine or combined azithromycin is not recommended for COVID-19 patients base on China’s New Coronavirus Pneumonia Diagnosis and Treatment Plan (Trial Version 8).

#### Convalescent plasma

Passive immunization, a technique to achieve immediate short-term immunization, to against infectious agents by administering pathogen-specific antibodies [62]. Human blood was also identified as a source of antibodies [63, 64]. Convalescent blood products obtained by collecting whole blood or plasma from patients who has survived in previous infection and produce humoral immune against the disease [65]. Convalescent plasma is widely used after large-scale epidemics of various viruses, such as MERS and Ebola virus (EBOV) [66, 67]. The results of a phase I clinical trial showed that 10 patients with severe COVID-19 pneumonia had all symptoms, especially fever, cough, shortness of breath, and chest pain, improved within 3 days after infusion of plasma from recovered patients, and laboratory tests, Imaging performance and viral load have improved significantly [68]. In addition, convalescent plasma is suitable for patients with rapid disease progression, severe and critically ill patients base on China’s New Coronavirus Pneumonia Diagnosis and Treatment Plan (Trial Version 8). The infusion dose needs to be determined based on the patient’s clinical condition and weight, usually the infusion dose is 200–500 ml.

#### Corticosteroids

A prospective meta-analysis of clinical trials of critically ill patients with COVID-19 showed that administration of systemic corticosteroids, compared with usual care or placebo, was associated with lower 28-day all-cause mortality [69]. Nowadays, According to China’s New Coronavirus Pneumonia Diagnosis and Treatment Plan (Trial Version 8), the patients with progressive deterioration of oxygenation indicators, rapid imaging progress, and excessive activation of the body’s inflammatory response, glucocorticoids should be used in a short period of time. The recommended dose is equivalent to 0.5 ~ 1 mg/kg/day of methylprednisolone, Meantime, WHO also made a strong recommendation for use of corticosteroids in severe and critical covid-19 because there is a lower risk of death among people treated with systemic corticosteroids [70].

#### Traditional Chinese medicine

Lianhuaqingwen capsule is an innovative patented traditional Chinese medicine for treating influenza. It has broad-spectrum antiviral, effective antibacterial, antipyretic and anti-inflammatory, cough and phlegm, and regulates immunity against viral respiratory infection equal effect [71]. More and more studies showed that Lianhuaqingwen capsule has antiviral and anti-inflammatory effects on the COVID-19 at the cellular level, which brings hope for the treatment of patients [71–73]. Lianhuaqingwen capsule has been approved by most countries for the prevention and treatment of light and common novel coronavirus pneumonia.

#### Other

##### NHC/EIDD-2801

β-D-N^4^-Hydroxycytidine (NHC/EIDD-2801) is an oral ribonucleoside analogue against various RNA viruses (including influenza, Ebola, CoV and Venezuelan Equine Encephalitis Virus (VEEV)) has broad-spectrum antiviral activity [74–76]. A study showed that the mice infected with COVID-19 or MERS-CoV, prophylactic and therapeutic administration of NHC/EIDD-2801 can improve lung function and reduce viral titer and weight loss [77]. The efficacy and oral bioavailability of NHC/EIDD-2801 against a variety of coronaviruses highlight their potential utility as effective antiviral agents against COVID-19 and other future zoonotic coronaviruses.

##### Tocilizumab

Tocilizumab is an immunosuppressive drug, mainly used to treat rheumatoid arthritis and systemic juvenile idiopathic arthritis. It is a humanized monoclonal antibody against interleukin 6 receptor (IL-6R). Previous studies have shown that patients infected with COVID-19 quickly activate inflammatory T cells and inflammatory monocytes / macrophages in the body, resulting in the significant increase in IL-6 levels in the blood, and the levels of IL-6 is associated with the mortality of patients with COVID-19 infection pneumonia [78]. A recent study showed that all patients experienced a recovery in body temperature after injection of Tocilizumab, and 90% of patients experienced a reduction in pulmonary symptoms. According to China’s New Coronavirus Pneumonia Diagnosis and Treatment Plan (Trial Version 8), Tocilizumab can be tried for patients with extensive lung disease and elevated IL-6 levels in the laboratory. The first dose of Tocilizumab is 4-8 mg/kg, and the recommended dose is 400 mg.

### Lung cancer patients awaiting treatment

For lung cancer patients who have not yet begun to medical treatment, the treatment strategy should be comprehensively considered baseed on the tumor burden and general situation of the patient. For early-stage lung cancer patients who waiting for surgery, the operation time should be postponed appropriately, especially for patients who mainly have ground glass shadow on the lungs in imaging, it has little effect on the overall condition in the short term. The specific situation should be based on specific condition to make a reasonable decision. For patients awaiting adjuvant chemotherapy after surgery, reasonable decisions should be made based on the postoperative pathology, clinical stage and prognostic indicators. A study showed that lung cancer patients who recover slowly from lung cancer surgery may still benefit from delayed adjuvant chemotherapy started up to 4 months after surgery [79]. Moreover, for patients with lymph node stage N2 after surgery with EGFR gene mutations or the rearrangements of ALK, oral targeted therapy drugs at home may be considered as the optional adjuvant treatment option, which may reduce side effects of chemotherapy and the cross infection caused by repeated hospital visits [80].

### Lung cancer patients receiving chemotherapy

Lung cancer patients undergoing chemotherapy may not be able to receive timely chemotherapy during the epidemic. The main concern in these patients is the development of tumor due to ingenuity of chemotherapy. Therefore, while we attach importance to the adverse effects of this epidemic on chemotherapy delays in lung cancer patients, we should also regard the adjustment of chemotherapy regimen rationally.

We recommend the patients who with a low tumor burden, stable disease, and receiving postoperative adjuvant chemotherapy and maintenance treatment can appropriately postpone inpatient chemotherapy or switch to oral chemotherapy targeted at home. For patients who require further chemotherapy, it is recommended that individual treatment under the guidance of an oncologist.

### Lung cancer patients receiving targeted therapy

Targeted therapies are revolutionized therapeutics which interfere with specific molecules to block cancer growth, progression, and metastasis [81]. Targeted therapy is recommended for patients with advanced lung cancer with the mutations of targeted driver EGFR, BRAF or the rearrangements of ALK or ROS1 [82]. However, we must be alert to the increase of adverse events that may be caused by targeted therapies in the current epidemic situation. Lung cancer patients who take oral targeted therapy drugs and have stable conditions should maintain the original drug treatment during the epidemic. Patients whose condition improved markedly after targeted therapy and those with stable disease can be appropriately deferred to the hospital for review during the epidemic. Although patients can be treated at home with oral drugs, attention should still be paid to the side effects and adverse events of some targeted drugs. Further, it is necessary to promptly consult an oncologist under proper protection under emergency symptoms or obvious disease progression.

### Lung cancer patients receiving immunotherapy

Immunotherapy has become an innovative technology for lung cancer therapy with the discovery of immunological checkpoints. It has shown great potential in a variety of advanced cancer treatments [83]. Drug binds to PD-1 molecule and generate steric hindrance that prevents PD-L1 from binding to PD-1 molecule, activates the immune response of PD-1 pathway-mediated, including anti-tumor immune response [84]. We suggest that for lung cancer patients who receiving immunotherapy during the epidemic, it is not urgent to receive immunotherapy on a set date. Considering the adverse events of pulmonary event caused by immunotherapeutic drugs, immunotherapy can be suspended or postponed in patients with stable disease.

## Management of adverse events

Some lung cancer patients who receiving antitumor treatment such as chemotherapy, targeted therapy, and immunotherapy need deal with treatment-related adverse events outside the hospital during the epidemic. The common adverse events include nausea, vomiting, and myelosuppression.

In order to reduce the case of nausea and vomiting, we recommend that patients maintain a reasonable diet and a good mood before antitumor treatment. Some studies showed that psychological adjustments such as listening to music, enjoying pictures, and yoga can also reduce the case of nausea and vomiting [85, 86]. If vomiting presists, a lateral position is necessary to prevent choking. After vomiting, we recommend that patients rinse their mouths with warm water and take an appropriate amount of saline to maintain electrolyte balance. In addition, if symptoms continue including abnormal vomiting color, excessive vomiting and dizziness, the patient should be taken to the hospital for symptomatic treatment.

Routine blood examination should be continue in the management of lung cancer patients during antitumor treatment to monitor the extent of myelosuppression through the important concerns including platelets, leukocyte counts, neutrophils, and red blood cells and hemoglobin levels. Mild to moderate myelosuppression can be treated with corresponding oral drugs. However, there are some conditions need to be treated under professional doctor guidance: neutrophil count < 1.0 × 10^9^/L, leukocyte count < 2.0 × 10^9^/L, platelet count < 50 × 10^9^/L, hemoglobin level < 100 g/L, or infection symptoms, or the occurrence of gum/nose bleeds and skin congestion.

## Discussion and conclusion

At present, COVID-19 has spread around the world. In deaths, the majority of patients over 60 years age and suffer from underlying diseases such as cancer [42]. Some studies have shown that about half of the survivors of malignant tumors have moderate fear of cancer recurrence [87]. Meantime, Fujita et al. found that 9.1% of lung cancer patients suffered anxiety and requested a treatment delay during the COVID-19 pandemic [88]. Coupled with the epidemic, lung cancer patients may more worry about the impact of COVID-19 and the delay of anti-tumor treatmen, which makes them more susceptible to illness, anxiety, depression and insomnia. Calvo et al. found that health surveillance and monitoring is an important part of maintaining wellbeing during the COVID-19 pandemic [89]. Another study also showed that a higher frequency of receiving COVID-19 information and news was associated with a lower risk of anxiety [90]. It is recommended high risk patients be identified for psychological morbidities and screening be improved to provide quick, cost effective psychological interventions via online platforms to manage symptoms during the COVID-19 pandemic [91]. In addition, considering the lack of time and resources for personalized screenings, We recommended programs such as Mental Health and Dynamic Referral for Oncology (MHADRO) to assist with screening for mental health problems for cancer patients [92]. Therefore, It is necessary to provide health services to cancer patients during COVID-19 pandemic, in order to prevent the predictable decline of cancer conditions, and prevent health system overload and health control crisis.

In this review, we focus on the epidemiological characteristics, early diagnosis and patient management of lung cancer patients during the COVID-19 epidemic. We provide helpful advice for the alternative transitional treatment and clinical management for lung cancer patients during the epidemic.

## Data Availability

Not applicable.
